# Subpopulation Treatment Effect Pattern Plot (STEPP) analysis of Ki67 assay according to histology: prognostic relevance for resected early stage ‘pure’ and ‘mixed’ lobular breast cancer

**DOI:** 10.1186/s13046-016-0325-z

**Published:** 2016-03-22

**Authors:** Luisa Carbognin, Isabella Sperduti, Matteo Brunelli, Lisa Marcolini, Rolando Nortilli, Sara Pilotto, Ilaria Zampiva, Sara Merler, Elena Fiorio, Elisa Filippi, Erminia Manfrin, Francesca Pellini, Franco Bonetti, Giovanni Paolo Pollini, Giampaolo Tortora, Emilio Bria

**Affiliations:** Medical Oncology, University of Verona, Azienda Ospedaliera Universitaria Integrata, P.le L.A. Scuro 10, 37124, Verona, Italy; Biostatistics, Regina Elena National Cancer Institute, Rome, Italy; Department of Pathology and Diagnostic, University of Verona, Azienda Ospedaliera Universitaria Integrata, Verona, Italy; Chirurgia ‘A’, Department of Surgery and Oncology, University of Verona, Azienda Ospedaliera Universitaria Integrata, Verona, Italy

**Keywords:** Lobular, Breast cancer, Ki67

## Abstract

**Background:**

The aim of this analysis was to investigate the potential impact of Ki67 assay in a series of patients affected by early stage invasive lobular carcinoma (ILC) undergone surgery.

**Methods:**

Clinical-pathological data were correlated with disease-free and overall survival (DFS/OS). The maximally selected Log-Rank statistics analysis was applied to the Ki67 continuous variable to estimate appropriate cut-offs. The Subpopulation Treatment Effect Pattern Plot (STEPP) analysis was performed to assess the interaction between ‘pure’ or ‘mixed’ histology ILC and Ki67.

**Results:**

At a median follow-up of 67 months, 10-years DFS and OS of 405 patients were 67.8 and 79.8 %, respectively. Standardized Log-Rank statistics identified 2 optimal cut-offs (6 and 21 %); 10-years DFS and OS were 75.1, 66.5, and 30.2 % (*p = 0.01*) and 84.3, 76.4 and 59 % (*p = 0.003*), for patients with a Ki67 < 6 %, between 6 and 21 %, and >21 %, respectively. Ki67 and lymph-node status were independent predictor for longer DFS and OS at the multivariate analysis, with radiotherapy (for DFS) and age (for OS). Ki67 highly replicated at the internal cross-validation analysis (DFS 85 %, OS 100 %). The STEPP analysis showed that DFS rate decreases as Ki67 increases and those patients with ‘pure’ ILC performed worse than ‘mixed’ histology.

**Conclusions:**

Despite the retrospective and exploratory nature of the study, Ki67 was able to significantly discriminate the prognosis of patients with ILC, and the effect was more pronounced for patients with ‘pure’ ILC.

**Electronic supplementary material:**

The online version of this article (doi:10.1186/s13046-016-0325-z) contains supplementary material, which is available to authorized users.

## Background

Invasive lobular carcinoma (ILC, 5 to 15 % of all invasive breast tumors), represents the second most common type of breast cancer after invasive ductal carcinoma (IDC), otherwise referred as invasive carcinoma of no special type [1, 2]. Recently, several evidences are supporting the concept that ILC belongs to a distinct family of breast disease with peculiar clinical and molecular characteristics [3, 4]. Indeed, ILC is more commonly associated with older age at presentation, larger tumor size and higher frequency of multifocality and bilaterality compared with IDC [1, 5].

With regard to molecular features, the most frequent genetic alteration of ILC is the loss of 16q chromosome, where the E-cadherin gene (*CDH1*) is located [6]. The loss of E-cadherin, a transmembrane glycoprotein that mediates the adhesion between the epithelial cells, promotes invasion and metastatic behavior and is also associated with the process of epithelial to mesenchymal transition [5].

Invasive lobular breast cancer differs from IDC for clinical outcome and recurrence pattern as well. It was historically considered a good prognostic subtype, given the more favorable pathological parameters (higher estrogen receptor [ER] expression, lower histological grade and lower mitotic index in comparison with IDC) [7, 8]. However, more recent findings suggest that, despite a trend towards slightly better short-term outcome, the overall prognosis for patients with ILC seems to be similar or worse than those with IDC, with a trend to progressively relapse more frequently at approximately 6 years after the diagnosis [1, 9, 10].

Thus, the prognosis of ILC still remains controversial and the definition of prognostic factors represents a critical issue for clinical practice. Overall, the tumor proliferation, measured by the immunohistochemical assessment of Ki67 antigen, a nuclear protein expressed in all cell-cycle phases other than G_0_, together with hormone receptor and human epidermal growth factor receptor 2 (HER2) status, stage and histological grade, represent the main accepted prognostic factors for early breast cancer [11].

Consistent data showed a statistically significant association between Ki67 level and prognosis [12], but the cut-offs to distinguish between ‘high’ and ‘low’ Ki67 varied from 14 to 29 %, also due to the continuous distribution of the variable and to analytic and pre-analytic barriers to standardized assessment. This variability does not allow to easily compare the results of studies and to speculate about the reliance and consistency between them [13].

As the majority of prognostic factors for early ILC, the value of Ki67 derives from the context of trials where the most of patients included are affected by IDC, thereby limiting its clinical utility in the specific context of ILC [14]. Thus, the long-term prognostic role of Ki67 in ILC has not yet been fully established.

The purpose of the current analysis was to investigate the potential long-term impact of Ki67 assay and the best prognostic cut-off value in a series of patients affected by early stage ILC undergone surgical treatment.

## Methods

### Patients’ population

Clinical charts of consecutive patients affected by early stage ILC undergone surgery at the University Hospital of Verona, between January 1990 and December 2013 were considered eligible. Inclusion criteria were ‘pure’ or ‘mixed’ (ductal-lobular) ILC diagnosis (stage I-III), curative surgery and availability of clinical-pathological (age, Performance Status [PS], menopausal status, type of surgery, clinical stage, treatments, grading, Ki67, ER, progesterone receptor [PgR], and HER2 status) parameters. The study was approved by the local Ethics Committee (Prot. CESC n° 24163, May 20^th^, 2014).

### End-points

The aim of this analysis was to evaluate the impact of Ki67 assay on disease free survival (DFS) and overall survival (OS), in order to identify the best prognostic cut-off for patients with resected ILC. The DFS was defined by the time between diagnosis and local or distant recurrence and OS was defined by the time between diagnosis and death for any cause.

### Immunohistochemical analyses

Immunohistochemistry was performed on surgical specimens by automatic instrument (Bond, Menarini, Florence) using 4 μm formalin-fixed paraffin-embedded tissues and the following primary antibodies: Ki67 (MM1, Novocastra, New Castle, UK, 1:50), ER (Rabbit SP1, Thermo Sc. Labvision, Fremont, CA,1:50), PR (PgR 636, Dako, Carpinteria, CA, 1:150), and HER2 (Hercept Test, Dako, Milan). The immunohistochemical staining for HER2 was scored according to Food and Drug Administration criteria, and tumors with a 2+ staining were tested for *HER2* gene copy number by fluorescent in situ hybridization (FISH, *PathVysion, Abbott spa, Rome)*.

### Statistics

Descriptive statistic was used to summarize pertinent study information. Follow-up was analyzed and reported according to Shuster [15]. The maximally selected Log-Rank statistics analysis was applied to the Ki67 continuous variable in order to estimate the most appropriate cut-off values able to split patients into groups with different DFS probabilities [16]. Associations between variables and groups according to Ki67 were analyzed (Chi-square test). The hazard ratio (HR) and the 95 % Confidence interval (95 % CI) were estimated using the Cox univariate model [17]. A multivariate proportional hazard model was developed using stepwise regression (forward selection, enter limit and remove limit, *p = 0.10* and *p = 0.15*, respectively), to identify independent predictors of outcomes in the whole population and in patients with pure ILC (this subgroup analysis was specified a priori). The Harrell’s guidelines for the identification of the correct number of covariates were taken into account for the power analysis (the number of events should have be more than 10 times greater than the number of investigated predictors, so that the expected error from the Cox model would be less than 10 %) [18]. Survival was calculated by the Kaplan–Meier product limit method. The log-rank test was used to assess differences between subgroups. Significance was defined at *p* < 0.05. The SPSS (18.0), R (2.6.1), and MedCalc (14.2.1) licensed statistical programs were used for all analyses.

To address the multivariate model overfit and to validate the results, a cross-validation technique, which evaluates the replication stability of the final Cox model in predicting the outcomes, was also investigated [19–21]. This technique generates a number of simulation datasets (at least 100, each approximately 80 % of the original size), by randomly selecting patients from the original sample, to establish the consistency of the model across less-powered patient’ samples.

Finally, the Subpopulation Treatment Effect Pattern Plot (STEPP) analysis was performed to assess the interaction between histological subtypes and Ki67; we aimed to evaluate whether (and how much) the prognostic effect (in terms of absolute DFS difference between ‘pure’ lobular and mixed ductal-lobular) varies according to the Ki67 [22].

## Results

### Patients’ characteristics

Data from 405 patients with operable or locally advanced ILC, undergone surgery were gathered. Overall patients’ characteristics are listed in Table 1. The median patients age was 60 years (range 35–96 years), 115 (28.4 %) and 290 (71.6) were premenopausal and postmenopausal, respectively. The majority of patients (379) were ER positive (93.6 %) and PgR positive (335 patients, 82.7 %). With regard to the adjuvant treatment, hormonotherapy was administered to 85.4 % of patients, while chemotherapy was administered in 39.3 %. Among HER2-positive patients, 59.1 % of them received trastuzumab. One hundred-sixteen (28.6 %) recurrences and 86 (21.2 %) deaths did occur at a median follow-up of 67 months (range 1–396 months). Median DFS was 172 months (95 % CI 156–188), with a 5- and 10-year rate of 80.4 and 67.8 %, respectively. Median OS was 225 months (95 % CI 200–250), with a 5- and 10-year rate of 90.4 and 79.8 %, respectively.

**Table 1:** Patients’ characteristics

Category	Patients number (%)
Menopausal status	
Premenopausal	115 (28.4)
Postmenopausal	290 (71.6)
Performance status (ECOG)	
0	379 (93.6)
1	22 (5.4)
2	4 (1.0)
Type of surgery	
Tumorectomy	173 (42.7)
Quadrantectomy	83 (20.5)
Mastectomy	149 (36.8)
Sentinel lymph-node biopsy	
No	157 (38.8)
Yes	248 (61.2)
Histological subtype	
Pure lobular	290 (71.6)
Ductal-lobular	105 (25.9)
Others	10 (2.5)
T descriptor according to TNM [7° Edition]	
1	233 (57.5)
2	128 (31.6)
3	31 (7.7)
4	11 (2.7)
Lymph-node status	
Negative	234 (57.8)
Positive	155 (38.2)
Unknown	16 (4.0)
Estrogen receptor status	
Negative	12 (3.0)
Positive	379 (93.6)
Unknown	14 (3.4)
Progesterone receptor status	
Negative	40 (9.9)
Positive	335 (82.7)
Unknown	30 (7.4)
HER2 status	
Negative	259 (64.0)
Positive	20 (4.9)
Unknown	126 (31.1)
Histological grade	
1	67 (16.5)
2	155 (38.3)
3	56 (13.8)
Unknown	127 (31.4)
Vascular invasion	
Absent	211 (52.1)
Present	84 (20.7)
Unknown	110 (27.2)
Multifocality	
Absent	316 (78.0)
Present	83 (20.5)
Unknown	6 (1.5)
Adjuvant hormonotherapy	
No	59 (14.6)
Yes	346 (85.4)
Adjuvant chemotherapy	
No	245 (60.5)
Yes	160 (39.5)
Adjuvant radiotherapy	
No	132 (32.6)
Yes	259 (64.0)
Unknown	14 (3.4)

Legend – Table 1: *ECOG* Eastern Cooperative Oncology Group

### Maximally selected log-rank statistics analysis

**Fig. 1:**
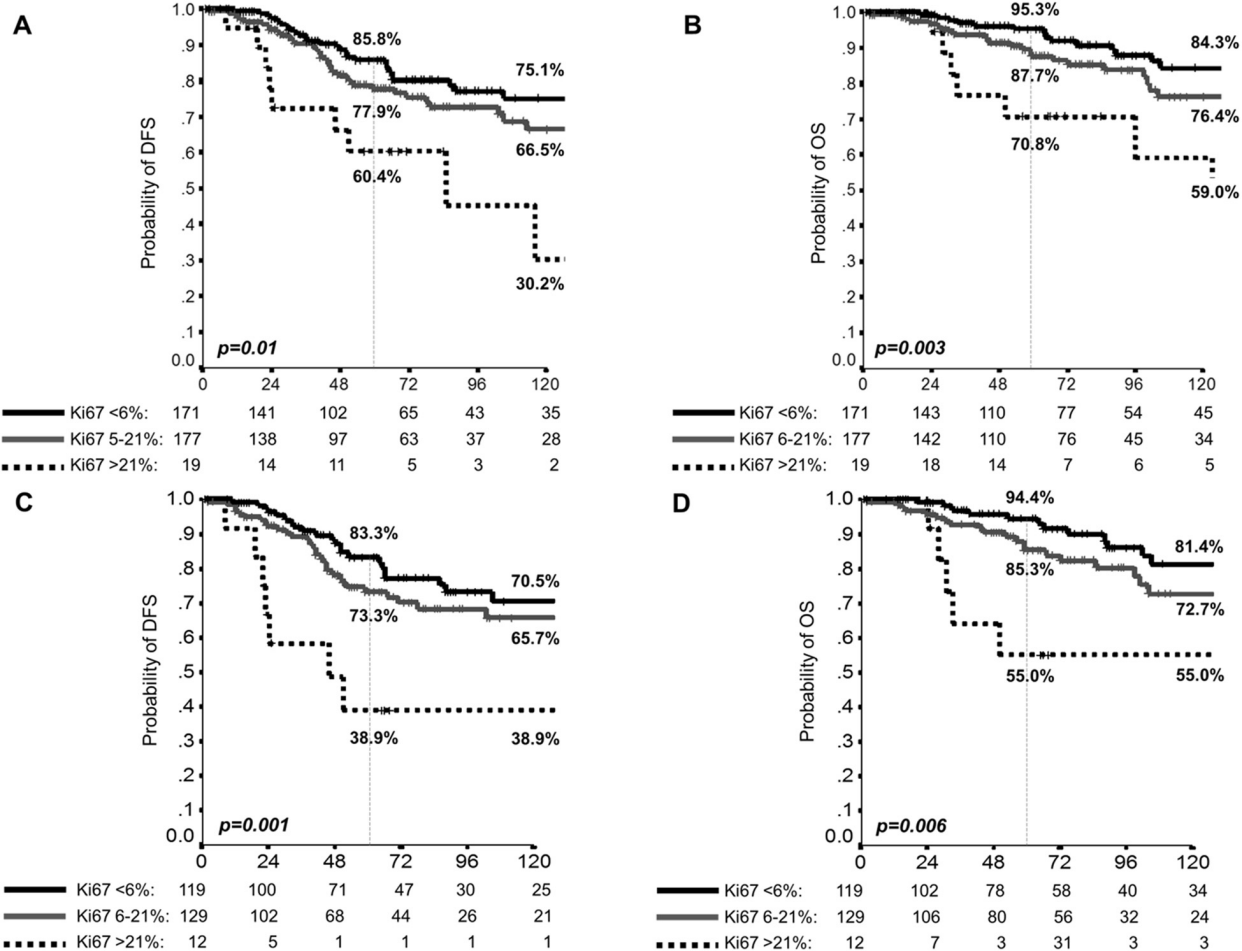
Disease-Free Survival (DFS) [Panel **a** and **c**] and Overall Survival (OS) [Panel **b** and **d**] according to Ki67, for patients with invasive lobular carcinoma (ILC) [Panels **a** and **b**] and patients with pure ILC [Panels **c** and **d**]; *p*-value: log-rank analysis

**Fig. 2:**
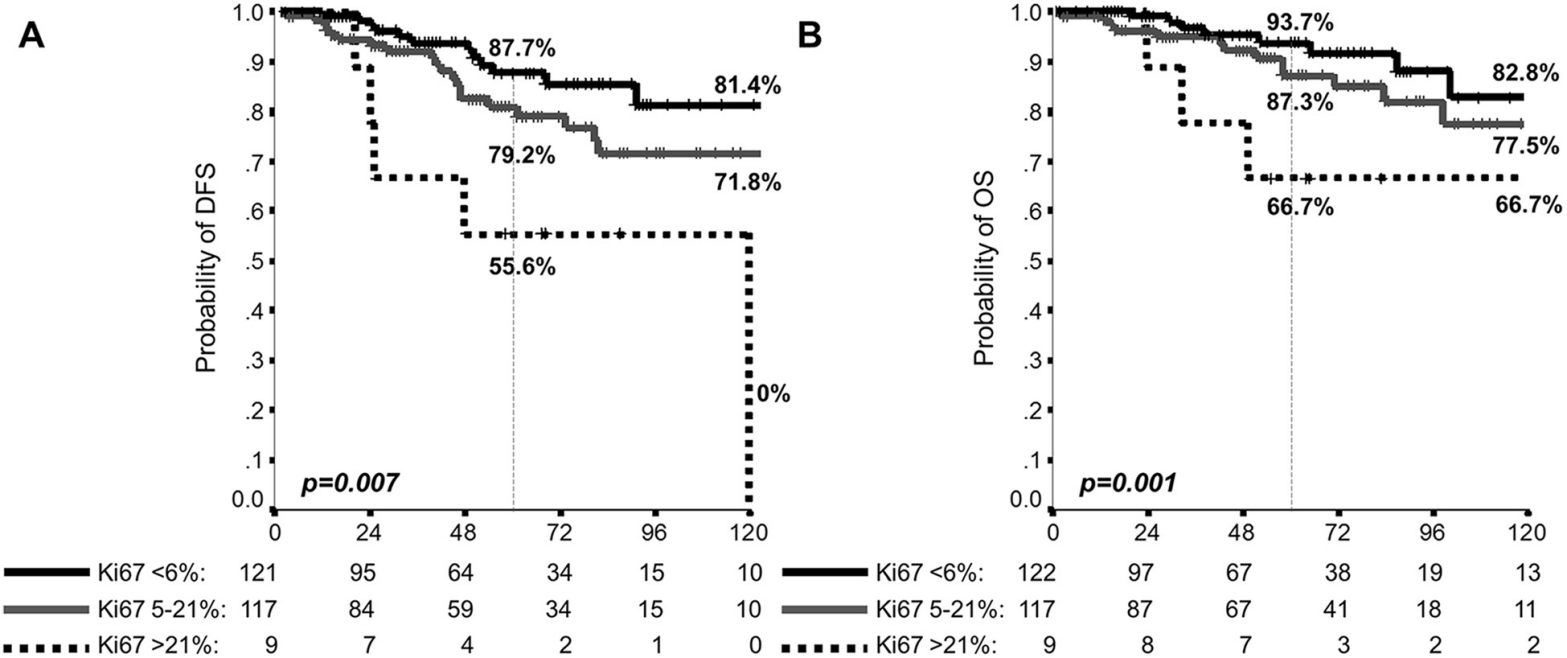
Disease-Free Survival (DFS) [Panel **a**] and Overall Survival (OS) [Panel **b**] according to Ki67, for patients with estrogen receptor positive/HER2-negative invasive lobular carcinoma (ILC); *p*-value: log-rank analysis

The optimal cut-off (absolute peak) in standardized Log-Rank statistics plot was 6 %. This identified value allowed to identify patients with a very good prognosis; thus, a further maximally selected Log-Rank statistics analysis was performed, by excluding patients with Ki67 < 6 %, in order to better stratify the prognosis of ILC. According to this analysis, the Ki67 cut point corresponds to 21 %. Patients characteristics and their differences according to groups identified by both cut-offs are reported in Table 2. These cut-offs significantly correlated with both DFS (Fig. 1, Panel a) and OS (Fig. 1, Panel b); outcomes according to Ki67 cut-offs of 6 % or 21 % are shown in Additional file 1: Figure S1. In order to further enhance the usefulness of the analysis and to generate implications for clinical practice, the DFS and OS according to Ki67 in the context of patients with ER positive and HER2-negative disease is shown Fig. 2. With the same principle and for clinical speculation, DFS and OS according to Ki67 and nodal involvement are shown in Additional file 2: Figure S2.

**Table 2:** Patients’ characteristics according to Ki67 groups

Patients’ characteristics	Patients (%)			*p*-value
	Ki67 < 6 % [*N* = 171]	6 % < Ki67 ≤ 21 % [*N* = 177]	Ki67 > 21 % [*N* = 19]	
Menopausal status				
Premenopausal	50 (29.2)	47 (26.6)	9 (47.4)	0.16
Postmenopausal	121 (70.8)	130 (73.4)	10 (52.6)	
Performance status (ECOG)				0.35
0	163 (95.3)	162 (91.5)	18 (94.7)	
1–2	8 (4.7)	15 (8.5)	1 (5.3)	
Type of surgery				
Conservative surgery	120 (70.2)	113 (63.8)	10 (52.6)	0.20
Mastectomy	51 (29.8)	64 (36.2)	9 (47.4)	
Sentinel lymph-node biopsy				
No	92 (53.8)	105 (59.3)	14 (73.7)	0.20
Yes	79 (46.2)	72 (40.7)	5 (26.3)	
Histological subtype				
Pure lobular	119 (69.6)	129 (72.9)	12 (63.2)	0.72
Ductal-lobular	48 (28.1)	43 (24.3)	7 (36.8)	
Others	4 (2.3)	5 (2.8)	0 (0)	
T Descriptor according to TNM [7° Edition]				
1	115 (67.3)	93 (52.5)	6 (31.5)	0.01
2	43 (25.1)	60 (33.9)	11 (57.9)	
3	11 (6.4)	17 (9.6)	1 (5.3)	
4	2 (1.2)	7 (4.0)	1 (5.3)	
Lymph-nodes status				
Negative	111 (64.9)	92 (52.0)	10 (52.6)	0.06
Positive	56 (32.7)	74 (41.7)	9 (47.4)	
Unknown	4 (2.3)	11 (6.2)	0 (0)	
Estrogen receptor status				
Negative	6 (3.5)	3 (1.7)	2 (10.5)	0.29
Positive	164 (95.9)	173 (97.7)	17 (89.5)	
Unknown	1 (0.6)	1 (0.6)	0 (0)	
Progesterone receptor status				
Negative	20 (11.7)	14 (7.9)	5 (26.3)	0.14
Positive	144 (84.2)	157 (88.7)	13 (68.4)	
Unknown	7 (4.1)	6 (3.4)	1 (5.3)	
HER2 status				
Negative	124 (72.5)	118 (66.7)	11 (57.8)	0.01
Positive	4 (2.3)	12 (6.8)	4 (21.1)	
Unknown	43 (25.1)	47 (26.6)	4 (21.1)	
Histological grade				
1	47 (27.5)	20 (11.3)	0 (0)	<0.0001
2	57 (33.3)	87 (49.2)	9 (47.4)	
3	15 (8.8)	33 (18.6)	6 (31.6)	
Unknown	52 (30.4)	37 (20.9)	4 (21.1)	
Vascular invasion				
Absent	105 (61.4)	102 (57.6)	3 (15.8)	<0.0001
Present	26 (15.2)	45 (25.4)	10 (52.6)	
Unknown	40 (23.4)	30 (16.9)	6 (31.6)	
Multifocality				
Absent	131 (76.6)	137 (77.4)	16 (84.2)	0.48
Present	38 (22.2)	38 (21.5)	2 (10.5)	
Unknown	2 (1.2)	2 (1.1)	1 (5.3)	
Adjuvant hormonotherapy				
No	20 (11.7)	12 (6.8)	5 (26.3)	0.02
Yes	151 (88.3)	165 (93.2)	14 (73.7)	
Adjuvant chemotherapy				
No	111 (64.9)	102 (57.6)	1 (5.3)	<0.0001
Yes	60 (34.1)	75 (42.4)	18 (94.7)	
Adjuvant radiotherapy				
No	48 (28.1)	54 (30.5)	8 (42.1)	0.48
Yes	118 (69.0)	114 (64.4)	11 (57.9)	
Unknown	5 (2.9)	9 (5.1)	0 (0)	

Legend – Table 2: *N* number, *p-value* chi-square test, *ECOG* Eastern Cooperative Oncology Group

### Multivariate analysis

At the multivariate analysis, Ki67, negative lymph-node status and adjuvant radiotherapy were significant independent predictors for longer DFS. With regard to OS, Ki67, negative lymph-node status, and age <60 years were significant prognostic predictors (Table 3). With regard to the 290 patients with pure ILC, Ki67 (HR 4.04, 95 % CI 1.82–8.98, *p* = 0.001), and negative lymph-node status (HR 3.45, 95 % CI 2.08–5.72, *p* < 0.001) were significant independent predictors for longer DFS. With regard to OS, Ki67 (HR 3.92, 95 % CI 1.55–9.94, *p* = 0.004), negative lymph-node status (HR 3.55, 95 % CI 1.90–6.63, *p* < 0.001), and age <60 years (HR 2.51, 95 % CI 1.35–4.67, *p* = 0.004) were significant prognostic predictors. Survival curves according to Ki67 for pure ILC patients are shown in Fig. 1, Panels c-d.

**Table 3:** Multivariate analysis

	Disease-free survival			Overall survival		
Variables	HR	95 % CI	*p*-value	HR	95 % CI	*p*-value
Lymph-node status (*Positive vs negative*)	4.57	2.14–9.75	<0.0001	5.38	2.26–12.79	<0.0001
Ki67 *(>21 % vs ≤21 %*)	3.61	1.35–9.63	0.010	12.58	4.13–38.23	<0.0001
Radiotherapy (*No vs Yes*)	2.72	1.10–4.66	0.026	2.1	0.89–4.91	0.087
Age *(>60 year vs ≤60 year*)	-	-	-	3.74	1.53–9.13	0.004

Legend – Table 3: *HR* Hazard Ratio, *CI* confidence intervals, *vs* versus, *yrs* years

### Internal validation analysis

At the internal cross-validation analysis, Ki67, lymph-node status and radiotherapy were confirmed as independent factors for DFS (replication rate: 85, 100, and 55 %, respectively) and Ki67, lymph-node status, and age, for OS (replication rate: 100, 98, and 96 %).

### STEPP analysis

**Fig. 3:**
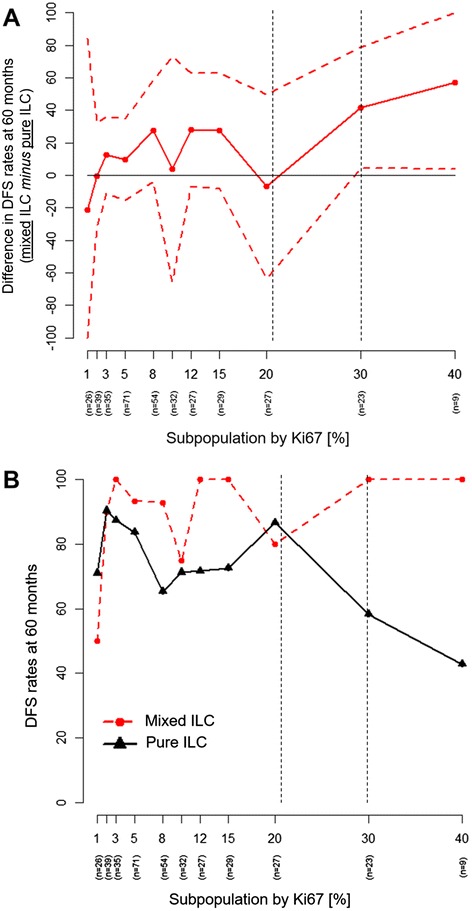
Subpopulation Treatment Effect Pattern Plot (STEPP) Analysis of Ki67 assay according to histology: Prognostic Relevance for resected early stage pure and mixed invasive lobular carcinoma (ILC); Panel **a**: differences in Disease-Free Survival (DFS) rates at 60 months, between patients with pure and mixed (ductal-lobular) ILC according to patients’ subpopulations clustered by Ki67 (%) [the solid line above 0 on the y-axis indicates better DFS at 60 months for mixed ILC compared with pure ILC as values of Ki67 increase from left to right on the x-axis]; Panel **b**: DFS rates at 60 months of patients with pure and mixed ILC according to patients’ subpopulations clustered by Ki67 (%)

The STEPP analysis shows a trend towards significant interaction according to histology (‘pure’ ILC versus mixed ductal-lobular ILC) in terms of both 5-years DFS differences (Fig. 3, Panel a) and absolute rates (Fig. 3, Panel b) when increasing Ki67 positivity (*p* = 0.03). Indeed, with high value of Ki67 patients with pure ILC show to perform worse than mixed histology in terms of DFS.

## Discussion

The results of the analysis reported herein suggest that the Ki67 assay is able to significantly discriminate the long-term prognosis of patients with primary resected ILC. Indeed, Ki67 emerges as a significant prognostic factor in multivariate analysis and the best cut-offs able to discriminate between very ‘good prognosis’ and ‘poor prognosis’ seems to be 6 and 21 %. If ‘very good performers’ (i.e. very low proliferation, Ki67 < 6 %) are excluded, the cut-off of 21 % is confirmed (and internal validated) as an independent predictor for longer DFS and OS.

The prognostic significance of Ki67, a well-established cell proliferation marker in cancer, have been extensively investigated in studies mainly conducted in IDC cases in order to provide early and accurate information upon both outcome and prediction of response to treatment. These studies have shown an independent significant association between high Ki67 expression and increased risk of breast cancer relapse and benefit of the addition of adjuvant cytotoxic chemotherapy [23, 24]. Thus, immunohistochemical Ki67 assay, together with ER, PgR and HER2 status, was chosen as a useful and easily-to-apply surrogate for gene expression profile to defined breast cancer molecular subtypes, despite a lesser analytical validity than molecular testing and a reliable clinical validation. This step represents a crucial issue for treatment decision of early breast cancer given that molecular subtypes support physicians in daily clinical practice.

Proliferation is a key process for breast cancer development, and these genes are among the most represented in the context of the currently validated genomic tests (ex. Oncotype Dx), which have been developed to assess the risk of recurrence and identify those patients who are most likely to benefit from chemotherapy [25, 26]. Although a standardized cut-off for Ki67 have not been established as the search for Ki67 cut-offs is unreliable and the assessment for Ki67 has very wide inter-laboratory variation [27], a threshold within the range of 20–29 % was considered indicative of high Ki67 status [11]. These evidences almost exclusively derived from patients with IDC, and these implications are applied in clinical practice for ILC patients as well.

To our knowledge, no previous study clearly demonstrated a significant prognostic effect of Ki67 on DFS or OS in the featured context of early ILC. Indeed, a study conducted in a large series of ‘pure’ ILC cases adopting a pre-determined Ki67 cut-off of 20 % demonstrated no independently significant prognostic role of Ki67 on prognosis [28]. Similarly, in another retrospective study the Ki67 value was a significant prognostic factor in univariate analysis, but did not reach significance after adjustment for others known prognostic factors [29].

With regard to histology, the multivariate analysis confirms that a high Ki67 (>21 %) is associated with poor prognosis, and the STEPP analysis suggests an interaction against ‘pure’ ILC, which display to have a worse prognosis in comparison with mixed ductal-lobular, as the Ki67 positivity increases. The aggressive biological behavior of high Ki67 ILC is also corroborated by the significant association between high Ki67 and other potential prognostic predictors in breast cancer, such as tumor size, lymph-node status, HER2 status, vascular invasion and histological grade (Table 2). However, only lymph-node status represents a significant independent predictor for survival at the multivariate analysis.

In the context of ILC, the magnitude of the benefit of the addition of chemotherapy to hormonal therapy represents an open question, in particular for the absence of prospective randomized trials [30]. In our study, the unfavorable prognostic trend of patients with high Ki67 is not counteracted by the adjuvant chemotherapy received by almost all these patients. However, the small sample size would not easily allow to detect the potential advantage of the addition of adjuvant chemotherapy to hormonal therapy in patients with hormone receptor positive and HER2 negative disease, considering that even in the best scenario of predominance of IDC this benefit is around 4 % [31].

Based on the STEPP analysis, the prognostic impact of high Ki67 on 5-year DFS is primarily driven by the very poor outcome of ‘pure’ ILC patients with highest levels of Ki67. The few number of these patients represents one of the crucial limitation of our study that allow only to generate a hypothesis concerning the prognostic role of Ki67 in ILC. These data open further perspectives for such histology, and deserve to be confirmed in larger series.

## Conclusion

Despite the retrospective and exploratory nature of the study, the different types and duration of adjuvant treatments, the absence of a central pathology review, our study indicates that Ki67 is able to significantly discriminate the prognosis of patients with ILC, and this effect is more pronounced for patients with pure ILC. In particular, if we derive suggestions for additional studies in the context of local laboratory values, the prognostic analysis of ILC according to Ki67 is able to identify patients with a low-proliferative tumor (Ki67 < 6 %, in general less than 5 %) and patients with a ‘true’ high-proliferative tumors (Ki67 > 21 %, higher than 20 %). It is important to emphasize that patients with a Ki67 of 21 % or higher represent a small sample size (about 5 %) of our whole ILC population. This data, although it is in line with previous studies that demonstrate the low mitotic index characterizing the most of LBC subtype, contributes to not draw any definitive conclusions and to not consider a Ki67 value of 21 % as an absolute cut-off. Moreover, the limited role of these cut-offs are also determined by the absence of standard Ki67 assessments’ methodology. Our results suggest to pay particular attention to those few LBC patients with a high Ki67 value as their prognosis could be closely affected by the high mitotic index. Certainly, all these results need to be validated in further studies before that the cut-offs can be used clinically. Moreover, studies including gene expression profiles are needed as well in order to clarify the biologic features of ILC.

## Ethics approval

The study was approved by the local Ethics Committee (Prot. CESC n° 24163, May 20^th^, 2014).

## Additional files

Additional file 1: Figure S1. Disease-Free Survival (DFS) [Panel A and C] and Overall Survival (OS) [Panel B and D], according to dichotomized Ki67 [Cut-off 6 %: Panel A-B; Cut-off 21 % Panel C-D] for patients with invasive lobular carcinoma (ILC); *p*-value: log-rank analysis. (TIFF 5811 kb) Additional file 2: Figure S2. Disease-Free Survival (DFS) [Panel A and C] and Overall Survival (OS) [Panel B and D] according to Ki67, for patients with lymph-nodes negative [Panel A and B] and lymph-nodes positive [Panel C and D] estrogen receptor positive/HER2-negative invasive lobular carcinoma (ILC); *p*-value: log-rank analysis. (TIF 386 kb)

## Abbreviations

DFS: disease free survival
ER: estrogen receptor
HER2: human epidermal growth factor receptor 2
IDC: invasive ductal carcinoma
ILC: invasive lobular carcinoma
OS: overall survival
PgR: progesterone receptor
PS: performance status
STEPP: Subpopulation Treatment Effect Pattern Plot
