# Supplementary figures and images for: Subpopulation Treatment Effect Pattern Plot (STEPP) analysis of Ki67 assay according to histology: prognostic relevance for resected early stage ‘pure’ and ‘mixed’ lobular breast cancer
Source: J Exp Clin Cancer Res. 2016 Mar 22;35:50. doi: 10.1186/s13046-016-0325-z (PMC4802900; doi:10.1186/s13046-016-0325-z)

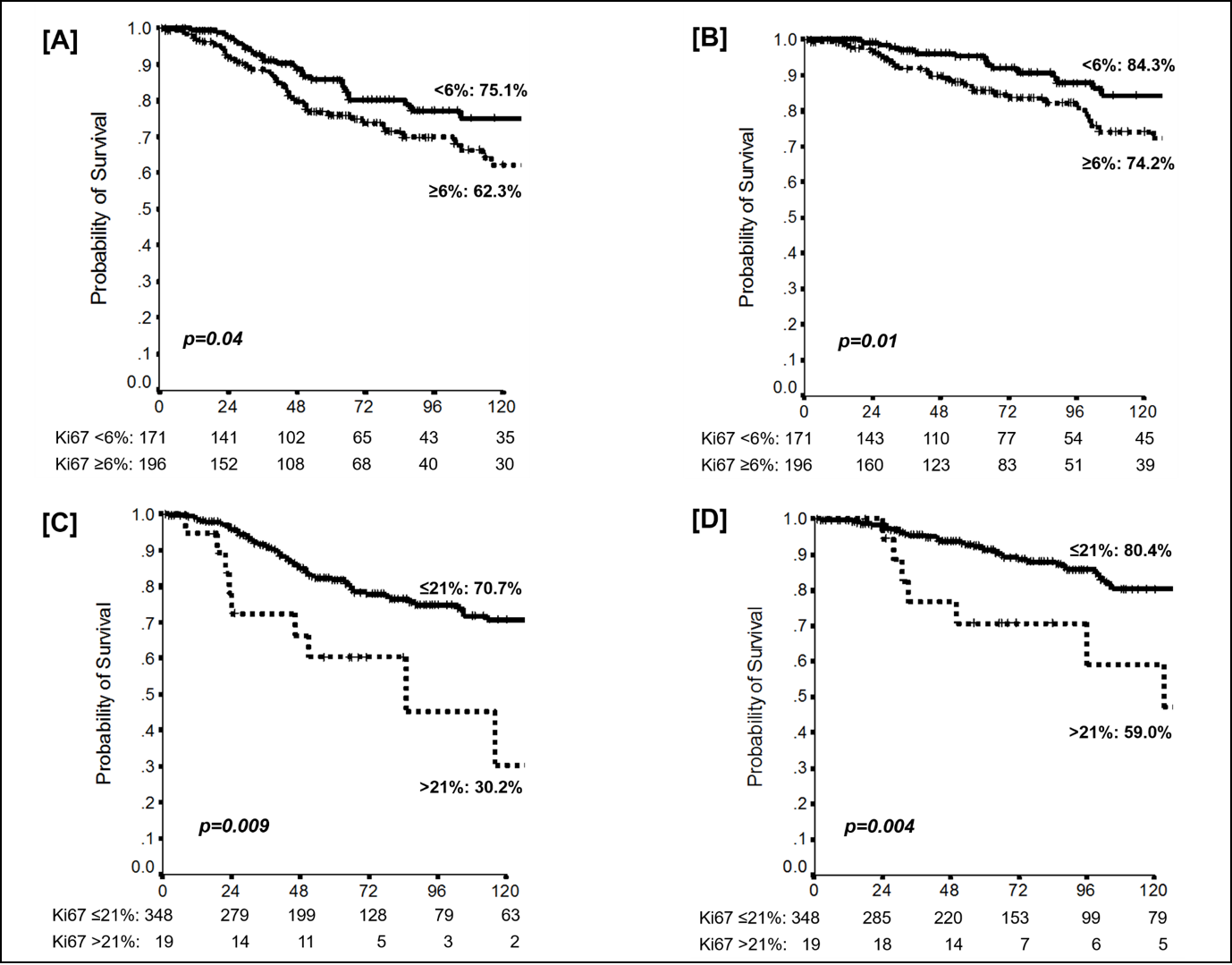

Supplement: Additional file 1: Figure S1. — Disease-Free Survival (DFS) [Panel A and C] and Overall Survival (OS) [Panel B and D], according to dichotomized Ki67 [Cut-off 6 %: Panel A-B; Cut-off 21 % Panel C-D] for patients with invasive lobular carcinoma (ILC); p-value: log-rank analysis. (TIFF 5811 kb) [file 13046_2016_325_MOESM1_ESM.tiff]

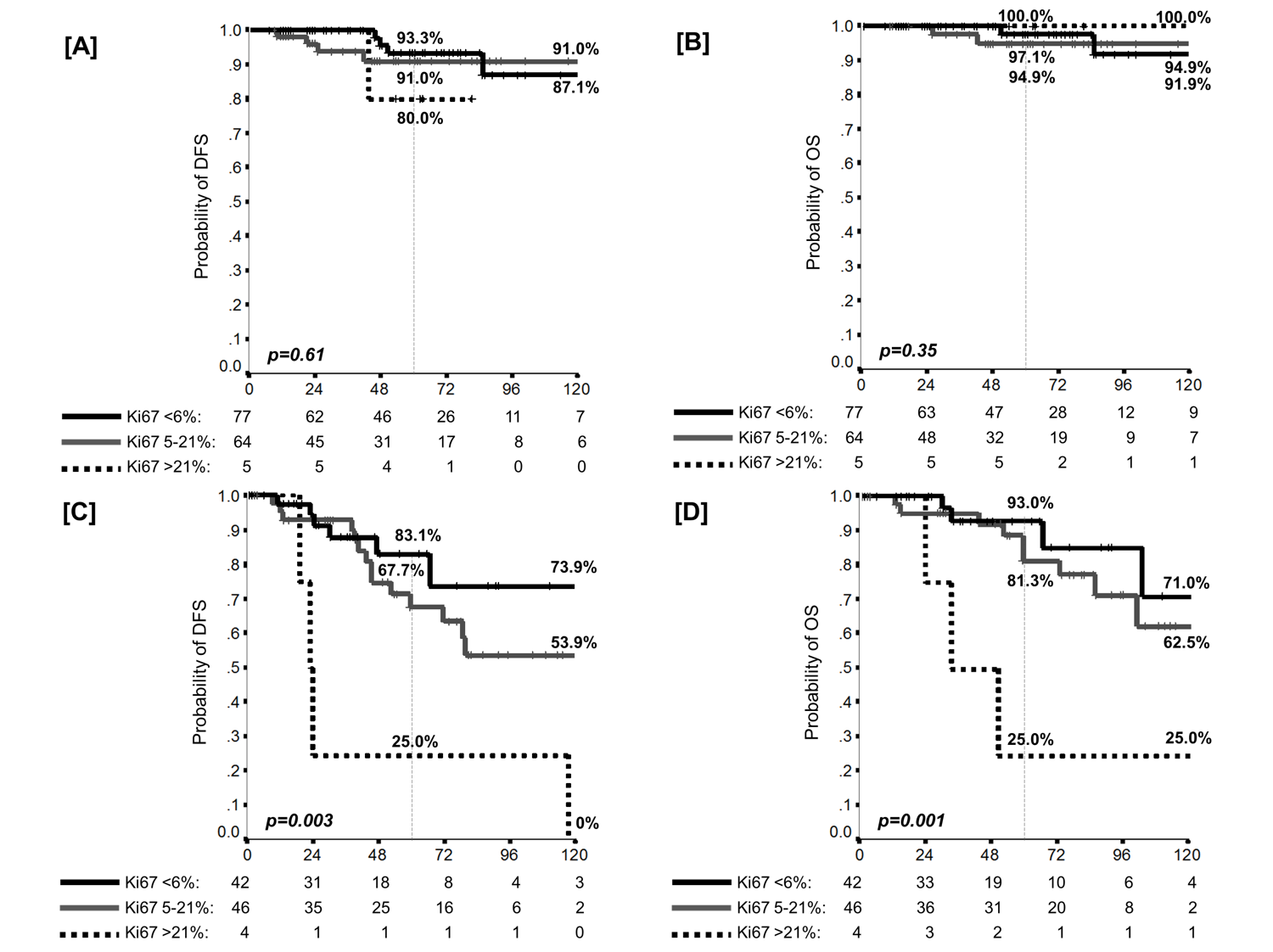

Supplement: Additional file 2: Figure S2. — Disease-Free Survival (DFS) [Panel A and C] and Overall Survival (OS) [Panel B and D] according to Ki67, for patients with lymph-nodes negative [Panel A and B] and lymph-nodes positive [Panel C and D] estrogen receptor positive/HER2-negative invasive lobular carcinoma (ILC); p-value: log-rank analysis. (TIF 386 kb) [file 13046_2016_325_MOESM2_ESM.tif]
